# RNAScope *in situ* Hybridization as a Novel Technique for the Assessment of c-KIT mRNA Expression in Canine Mast Cell Tumor

**DOI:** 10.3389/fvets.2021.591961

**Published:** 2021-02-16

**Authors:** Davide De Biase, Francesco Prisco, Giuseppe Piegari, Arianna Ilsami, Ilaria d'Aquino, Valeria Baldassarre, Federica Zito Marino, Renato Franco, Serenella Papparella, Orlando Paciello

**Affiliations:** ^1^Department of Veterinary Medicine and Animal Production, University of Naples Federico II, Naples, Italy; ^2^Department of Mental and Physical Health and Preventive Medicine, University of Study of Campania “Luigi Vanvitelli”, Naples, Italy

**Keywords:** RNAscope, c-kit, mRNA, immunohistochemistry, canine mast cell tumor

## Abstract

RNA is considered as an indicator of the dynamic genetic expression changes in a cell. RNAScope is a commercially available *in situ* hybridization assay for the detection of RNA in formalin-fixed paraffin-embedded tissue. In this work, we describe the use of RNAScope as a sensitive and specific method for the evaluation of *c-KIT* messenger RNA (mRNA) in canine mast cell tumor. We investigated the expression of *c-KIT* mRNA with RNAscope in 60 canine mast cell tumors (MCTs), comparing it with the histological grade and KIT immunohistochemical expression patterns. Our results showed an overall good expression of *c-KIT* mRNA in neoplastic cells if compared with control probes. We also observed a statistically significant correlation between histological grade and *c-KIT* mRNA expression. No correlations were found between KIT protein immunohistochemical distribution pattern and *c-KIT* mRNA expression or histological grade. Our results provide a reference basis to better understand *c-KIT* mRNA expression in canine MCTs and strongly encourage further studies that may provide useful information about its potential and significant role as a prognostic and predictive biological marker for canine MCTs clinical outcome.

## Introduction

Canine mast cell tumor (MCT) is one of the most common neoplastic disease in dogs accounting for ~20% of all canine skin tumors (1, 2). Cutaneous MCTs can have a very variable biological behavior (1), and several studies have focused on the investigation of predictive factors for MCTs outcome. These factors include the location of the tumor (3, 4), surgical margins (5–9), mitotic activity (10, 11), nuclear morphometry (12, 13), and vascular density (14). Immunohistochemical expression and protein localization of KIT in neoplastic cells is currently one of the most informative markers for prognostication of canine MCTs (1, 15–17). KIT is a tyrosine kinase surface growth factor receptor encoded by the proto-oncogene *c-KIT* that plays a central role in the survival, proliferation, differentiation, and migration of mast cells (15). It has been extensively described that the immunohistochemical localization of KIT protein in neoplastic canine mast cells may have three different patterns: perimembranous labeling (pattern I), focal or stippled cytoplasmic labeling along with loss of perimembranous labeling (pattern II), and diffuse cytoplasmic labeling (pattern III) (1, 15, 17, 18). Pattern I has been associated with a low aggressive biological behavior, whereas both patterns II and III have been associated with a decreased overall survival time and an increased incidence of local recurrence (16, 18, 19). As for now, there is no truly reliable biological marker that can predict canine MCT behavior (17), so it is of utmost importance to investigate novel biomarkers and assays that can be sensitive, specific, and predictive at the same time. RNA is an ideal indicator of the dynamic genetic expression changes in a cell; thus, it has recently emerged as a resource to discover novel biomarkers (20). Several authors have recently explored the role of *c-KIT* messenger RNA (mRNA) as a potential biological and prognostic marker for canine MCTs by assessing its expression levels by polymerase chain reaction (PCR) in blood or neoplastic tissue (21, 22). The results obtained from these studies have been so far elusive, and no significant correlation between *c-KIT* mRNA expression levels and other prognostic and predictive markers or clinical outcome was found (21, 22). The detection and measure of mRNA expression with traditional techniques that require the isolation of single cells from their native context, such as PCR, can mask the cell-to-cell variations in gene expression (23) and may result in the loss of important information especially on the spatial relationship of the analyzed cells (24). RNAScope is a commercially available RNA *in situ* hybridization (ISH) that allows visualization of single RNA molecules in individual cells in a variety of sample types including formalin fixed paraffin-embedded tissue (25). RNAScope has gained remarkable attention as a technology that detects alternative molecules to protein, and its use has been tested and validated in several pathologies in human medicine (26–30). Importantly, the major difference with the standard RNA ISH is that RNAScope detect target-specific probe minimizing nonspecific off-target signals, thus resulting in highly specific staining (27, 31). The aim of the current study was 2-fold: first, the assessment of feasibility of RNAScope in detecting and measuring *c-KIT* mRNA in formalin fixed paraffin-embedded (FFPE) tissue sections of canine MCTs; second, the investigation of correlation between *c-KIT* mRNA by RNAScope, histological grade, and KIT protein localization in canine MCTs.

## Materials and Methods

### Samples, History, and Histological Diagnosis

A retrospective cohort study has been performed on primary cutaneous MCTs submitted to the Diagnostic Service of the Pathology and Animal Health of the Department of Veterinary Medicine (University Federico II of Naples, Italy) from 2016 to 2019. The experiments were subsequent to the clinical informed consensus from the animal's owners and in compliance with the current national legal treatment of animal tissue samples.

Samples were fixed in 10% buffered formalin for no more than 4 days and embedded in paraffin, and 4-μm-thick sections were routinely stained with hematoxylin and eosin (HE) for histological evaluation and tumor grading. To decrease interobserver variation, histological grade of each tumor was confirmed according to the Kiupel histological grading system for canine cutaneous MCT (32) by two independent pathologists (OP and DDB). When grading differed, decision was taken by consensus. Kiupel two-tier histological grading system was chosen because it was demonstrated having a 96.8% interobserver consistency and it is predictive of overall survival (33). The anamnestic and clinical data such as breed, age, and sex were also collected.

### Tissue Microarray

Sixty MCTs were divided in two groups based on histological grade (low and high grade) and selected for immunohistochemistry and RNAScope. Cases were included in a tissue microarray (TMA) made up of 66 cores, with 60 cores from the selected cases and 6 from control tissues (4 skin biopsies and 2 canine testes with no pathological lesions). Testes were chosen as positive control tissue because it has been described that KIT protein may be normally observed in Leydig cells and in spermatogonia (34). A sector map consisting of an Excel sheet (Microsoft, Redmond, WA) was designed in order to depict the exact position of each case for each core sample within the tissue array (35). The layout was asymmetrically designed, and different control tissues were included on TMA both as a landmark (orientation core) and negative control. The area of interest was selected by light microscopy examination on the HE slides according to the following criteria: [1] the presence of a representative and highly cellular neoplastic area and [2] the absence of edema, necrosis, inflammation, and desmoplasia. Tissue microarray construction was based on the method of Kononen et al. (36). Briefly, a cylindrical core of paraffin wax-embedded block (the “donor”) was extracted manually with a skin biopsy punch of 2 mm in diameter and subsequently reintegrated in previously created empty cylinders (the “recipient”) (37). Five serial sections were cut from the TMA: the first was stained with HE as quality control to review the array, assess its quality (38), and confirm the presence of neoplastic tissue; the second was used to perform immunohistochemistry, and the other three were used to perform RNAScope assay, each one with the specific probe.

### Histology and Immunohistochemistry

Immunohistochemical staining for the evaluation of KIT staining patterns was performed using a horseradish peroxidase (HRP) method (39, 40). Briefly, 4-μm-thick sections of MCTs were mounted on a positively charged glass slides (Bio-Optica, Milan, Italy). Antigen retrieval pretreatments were performed using a heat-induced epitope retrieval (HIER) citrate buffer pH 6.0 (Bio-Optica, Milan, Italy) for 20 min at 98°C. Following, endogenous peroxidase (EP) activity was quenched with 3% hydrogen peroxide (H_2_O_2_) in methanol, and sections were blocked with a protein block (MACH1, Biocare Medical LLC, Concord, California, USA) for 30 min each. Slides were sequentially incubated overnight at 4°C with primary antibody diluted in phosphate-buffered saline (PBS) (0.01 M PBS, pH 7.2). Primary antibodies included polyclonal rabbit antihuman CD117/KIT diluted 1:300 (DAKO, Milan, Italy). Antibody deposition was visualized using the 3,3′-diaminobenzidine (DAB) chromogen diluted in DAB substrate buffer, and the slides were counterstained with hematoxylin. Between all incubation steps, slides were washed two times (5 min each) in PBS. In the corresponding negative control sections, the primary antibody was either omitted or replaced with a 1:20 dilution of rabbit serum (Code 011-000-120, Jackson Immuno Research, West Grove, PA, USA) according to the most recent and relevant guidelines (41). KIT immunohistochemical staining was evaluated as previously described for canine cutaneous MCTs by Kiupel et al. (18). Immunohistochemical scoring was independently performed by two pathologists (OP and DDB) with a concordance rate of 95%.

In brief, we identified three patterns of KIT protein localization:

1. KIT pattern I, defined by membranous labeling in >90% of neoplastic cells;
2. KIT pattern II, defined by focal perinuclear or stippled cytoplasmic labeling and loss of perimembranous labeling in >10% of neoplastic cells; and
3. KIT pattern III, defined by diffuse cytoplasmic labeling in >10% of neoplastic cells.

The MCTs chosen for this study presented at least 10% (estimated on the basis of 100 neoplastic cells in a high-power field) of the neoplastic cells with strong expression of KIT. Cells on the margins of the tissue sections were not considered due to possible artifactual staining.

### RNAScope mRNA *in situ* Hybridization Assay

**Figure 1:**
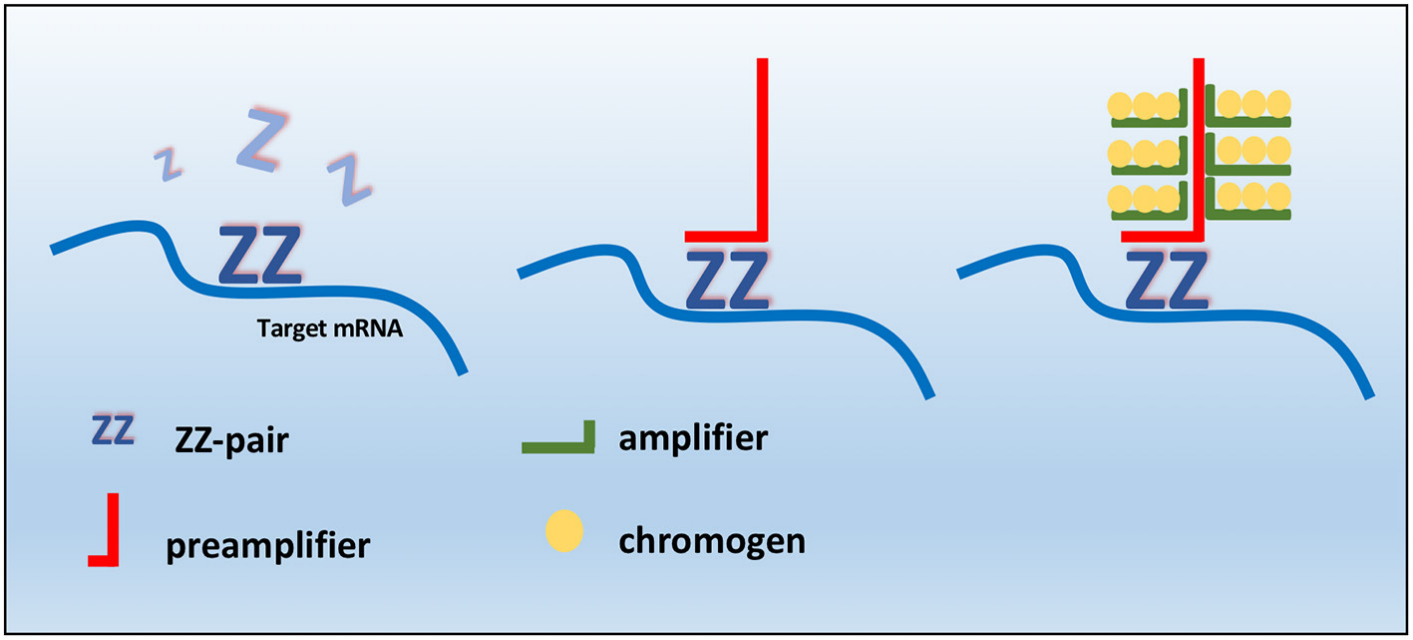
Schematic RNAscope assay procedure. RNAscope “ZZ” probe pairs bind the RNA target sequence, but a single Z probe is not sufficient to bind preamplifier. Two adjacent probes create a target for preamplifier molecules, which bind amplifiers that allow the probe-specific enzyme to bind to the chromogen.

Manual RNAscope assays was performed using BaseScope^TM^ v2 Assay (cod. # 322350, Bio-Techne, Milan, Italy) according to the manufacturer's protocol. The RNAScope assay consists of target probes and a signal amplification system composed of a preamplifier, amplifier, and label probe. A schematic RNAscope assay procedure is shown in Figure 1. In the first step, tissues are fixed, and permeabilized to allow the access of the target probe. In the second step, target RNA-specific oligonucleotide probes (conceptualized as a “Z”) are hybridized in pairs (“ZZ”) to multiple RNA targets. In a third step, the detection is carried out by specific binding of oligonucleotide preamplifier molecules linked to several amplifiers containing multiple chromogenic labels (27, 31, 42). In the last step, signals are detected by developing a chromogen to produce small punctate dots that can provide a quantitative and measurable result. Importantly, the preamplifier cannot bind to a single Z probe (non-paired Z probe) because a Z pair is necessary to bind the preamplifier and generate signals (42). Briefly, tissue sections were baked for 1 h at 60°C, deparaffinized, and treated with Pretreat 1 (Bio-techne, Milan, Italy) for 10 min at room temperature (RT). Target retrieval was performed for 15 min at 100–104°C, followed by protease treatment for 15 min at 40°C. Probes were then hybridized for 2 h at 40°C followed by RNAscope amplification followed by red chromogenic detection. TMAs were counterstained with hematoxylin and mounted with Bio-Mount (Bio-Techne, Milan, Italy). In this study, the following RNAscope probes were used: CI-KIT (cod. #512801, Bio-Techne, Milan, Italy) probe encodes for *cKIT* mRNA that may be detected both in the cytoplasm and nuclei, CI-PPIB (cod. # 437441, Bio-Techne, Milan, Italy) as positive control probe, and dihydrodipi-colinate reductase (dapB), a bacterial gene (cod. #310043, Bio-Techne, Milan, Italy) as negative control probe. PPIB, which encodes for a cyclosporine-binding protein (cyclophilin B), is expressed at a sufficiently low level in most tissues; hence, it is the recommended positive control (28). The stained slides of each sample were finally analyzed using the RNAscope manufacturer scoring system. RNAscope CI-KIT, positive control CI-PPIB, and negative control dapB probe signal results were categorized into five grades according to the following scoring guidelines: score 0, no staining or <1 dot for every 10 cells (visible at 40× magnification); score 1, 1–3 dots per cell (visible at 20–40 magnification); score 2, 4–10 dots per cell with very few dot clusters (visible at 20–40 magnification); score 3, >10 dots per cell with <10% positive cells having dot clusters (visible at 20× magnification); score 4, >10 dots per cell with more than 10% positive cells having dot clusters (visible at 20× magnification). The scoring of mRNA expression was independently performed by two pathologists (OP and DDB) with a concordance rate of 87%.

### Statistical Analysis

Statistics were computed using SPSS Version 22.0 (IBM Corporation, Armonk, NY, USA). The correlation between histological grade, *c-KIT* mRNA expression, and KIT immunohistochemical pattern was evaluated using Spearman's Rho correlation (Past 1923.10 software); *p* < 0.05 was considered statistically significant.

## Results

### Dogs' Demographics

A total of 60 dogs matched our selection criteria and were included in the study. The mean age of patients at presentation was 8 years (±2.8 SD). Males and females were similarly represented in our cohort, with 29 female (14 spayed, 15 intact; 48.2 and 51.8%, respectively) and 31 male (11 neutered, 20 intact; 35.4 and 64.6%, respectively) dogs. The selected cases represented 12 breeds including mixed breed dogs (*n* = 27, 45%), Labrador retriever (*n* = 9, 15%), Sharpei (*n* = 3, 5%), Boxer (*n* = 3, 5%), Setter (*n* = 5, 8.3%), and 9 other breeds that were represented by single animals (Golden retrievers, Pug, Bernese mountain dog, Caucasian shepherd, Beagle, Yorkshire, Pinscher, Dachshund, Staffordshire bull terrier).

### Histology and Immunohistochemistry

**Figure 2:**
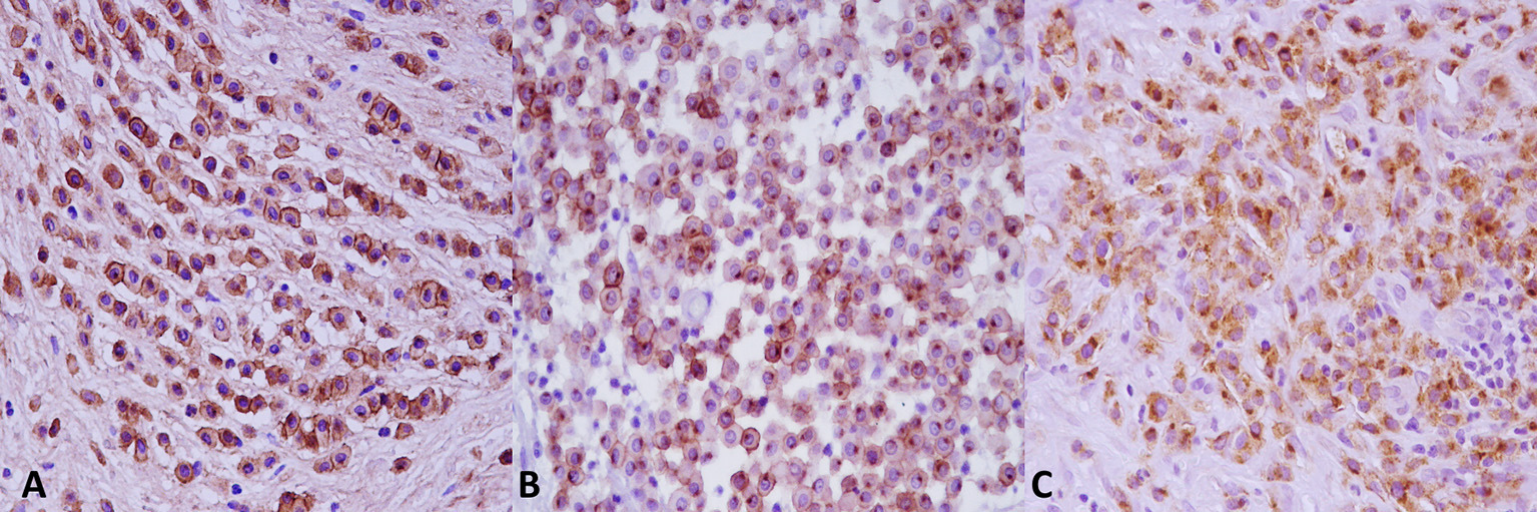
KIT immunostaining patterns. **(A)** Pattern 1, membrane associated. **(B)** Pattern 2, cytoplasmic focal (paranuclear). **(C)** Pattern 3, cytoplasmic diffuse. Anti-KIT antibody immunohistochemistry, 3,3′-diaminobenzidine (DAB) chromogen, hematoxylin counterstain. Original magnification, 40×.

Based on Kiupel two-tier grading system, 36 tumors (60%) were identified as low-grade MCTs, while the remaining cases (*n* = 24, 40%) were graded as a Kiupel high-grade MCTs. The agreement between the two pathologists for grading was good (k coefficient = 0.83). Histologically, low-grade MCTs were unencapsulated, poorly circumscribed neoplasms, and showed a moderately to highly cellular proliferation composed of sheets or cords of neoplastic mast cells that infiltrated the dermis, often elevating the epidermis and separating adnexal structures and fibers and collagen bundles (Supplementary Figure 1A). Neoplastic cells were round with distinct cell borders, moderate amounts of amphophilic cytoplasm occasionally containing fine basophilic granules and generally centrally located, and round nuclei with coarsely stippled chromatin. Scattered throughout the neoplasm were moderate to high numbers of eosinophils (Supplementary Figure 1B). Multifocally, there were areas of edema, necrosis, and hyalinized collagen (*flame figures*) (Supplementary Figure 1C). Mitotic figure average was 1 for 10 high power fields (HPFs). High-grade MCTs were highly cellular, usually not encapsulated and infiltrative. Tumor cells showed severe atypical cytological features such as karyomegaly, anisokaryosis, and a high number of mitotic figures (at least 7 in 10 HPFs). Multinucleated cells were also observed, often in proximity of degenerate collagen fibers (Supplementary Figure 1D). Collagenolysis, sclerosis, necrosis, and moderate to severe, diffuse, eosinophilic inflammatory infiltrates were also found. By immunohistochemistry, the prevalence of each pattern of KIT localization in the two groups (low vs. high histological grade) is summarized in Table 1 KIT immunostaining patterns. The three different KIT immunohistochemical patterns are depicted in Figure 2. As expected, immunolabeling of KIT was rarely observed in mast cells scattered in normal skin biopsies, whereas the immunoreactivity was moderate to high in Leydig cells and spermatogonia in normal testicular parenchyma.

**Table 1:** Number of cases for each immunohistochemical KIT protein pattern and RNAScope score.

	**Histological grade**	
	**Low**	**High**
**KIT expression pattern**		
1	13	3
2	13	15
3	9	5
No staining	1	1
**RNAscope score**
0	23	1
1	8	1
2	1	3
3	2	3
4	2	16

### RNAScope

**Figure 3:**
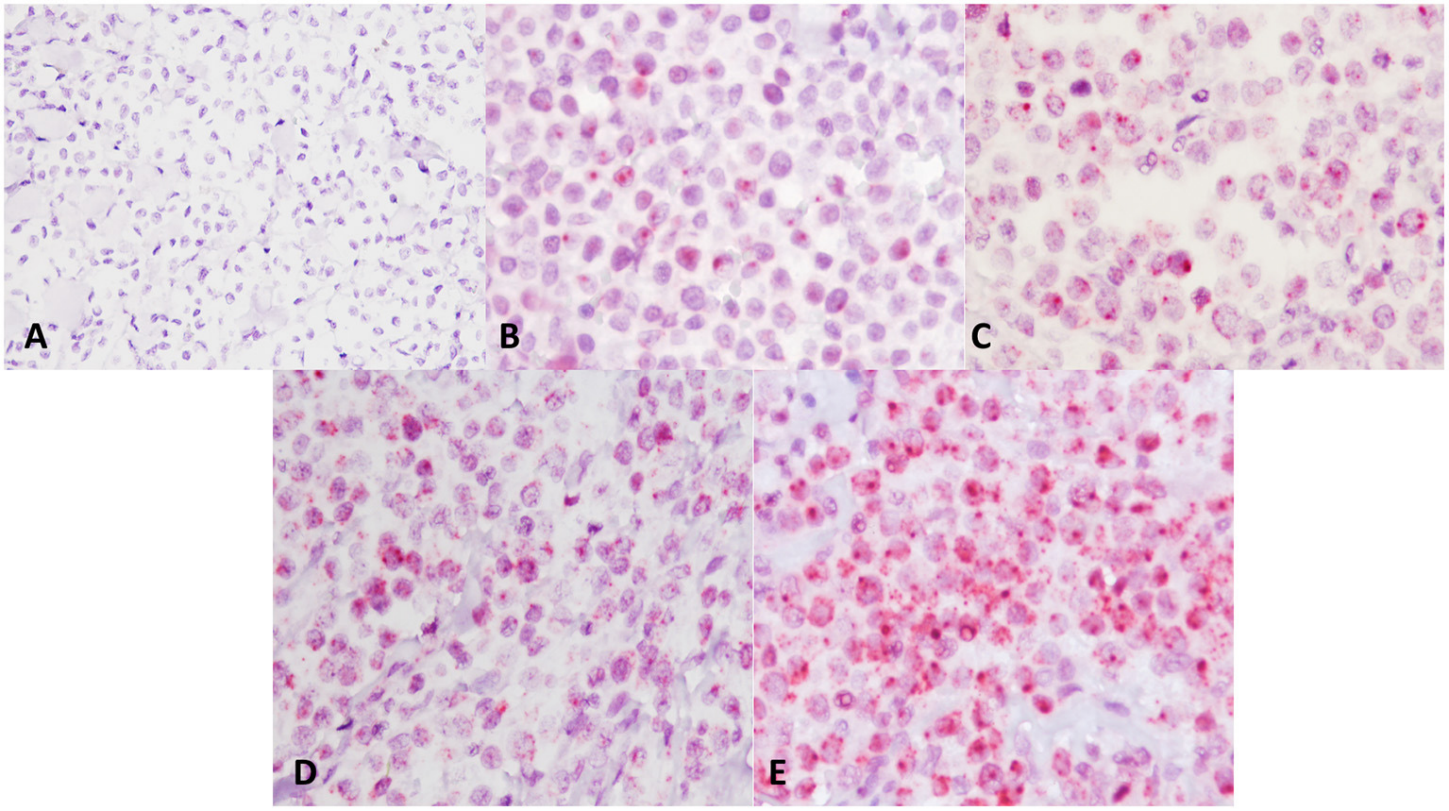
*c-KIT* messenger RNA (mRNA) expression in mast cell tumors samples and distribution of scores. Representative microphotographs show c-KIT mRNA expression as a microdotted staining pattern in the cytoplasm and nuclei of neoplastic cells: **(A)** score 0, **(B)** score 1, **(C)** score 2, **(D)** score 3, and **(E)** score 4. Hematoxylin counterstain. Original magnification, 40×.

**Figure 4:**
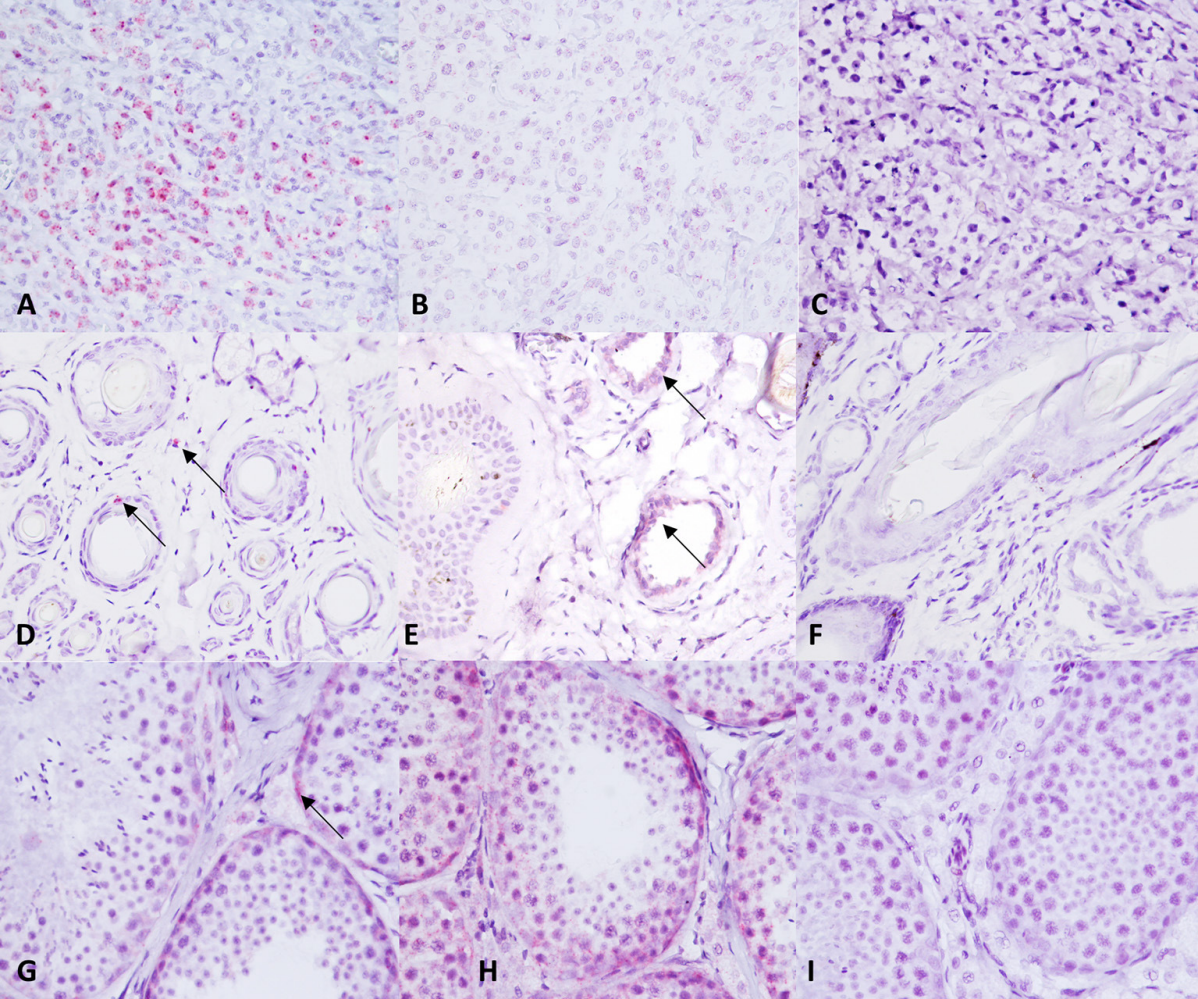
*c-KIT* and control probes expression in control tissue samples. **(A–C)** c-KIT, PPBI, and dihydrodipi-colinate reductase (dapB) expression in a canine mast cell tumor. **(D–F)** c-KIT, PPBI, and DapB expression in a normal canine skin biopsy. **(G–I)** c-KIT, PPBI, and DapB expression in a normal canine testis. Hematoxylin counterstain. Original magnification, 40×.

TMA sections were evaluated by RNAscope for expression of c-KIT mRNA, positive control probe PPIB, and negative control probe DapB. In MCT samples, *c-KIT* mRNA signals had a dotted hybridization pattern, and they were exclusively located within the cytoplasm and nuclei of neoplastic cells. A semiquantitative assessment of *c-KIT* mRNA expression was performed and scored; the scores for each group (low vs. high histological grade) is summarized in Table 1. Representative microphotographs of each staining score are shown in Figure 3. Consecutive serial section samples hybridized with PPBI and DapB mRNA probes showed, respectively, a low to moderate and an absent signal in neoplastic cells if compared to c-KIT mRNA probe (Figures 4A–C). As for control tissues, in normal skin, we observed no signal *of c-KIT* mRNA and a low to moderate signal of PPBI probe, but no signal of DapB control probe was observed (Figures 4D–F). In testicular parenchyma, we observed a moderate signal of c-KIT mRNA in Leydig cells and spermatogonia and a high signal of PPBI probe and no signal of the negative DapB probe (Figures 4G–I).

A statistically significant correlation was observed between histological grade and *c-KIT* mRNA expression (Spearman *r* = 0.7418; *p* < 0.0001): low-grade MCTs showed an absent or a weak expression of *c-KIT* mRNA; and high-grade MCTs showed an increased number of dots and dot clusters indicating a higher c-kit mRNA expression. No statistically significant correlation was found between histological grade and immunohistochemical KIT pattern (Spearman *r* = 0.1747; *p* = 0.1818) or between *c-KIT* mRNA expression and immunohistochemical KIT pattern (Spearman *r* = 0.1545; *p* = 0.2387). Correlation matrix comprising both Spearman r and p value for each pair of variables is summarized in Table 2. Statistical results are also depicted as a heat map of the correlation matrix.

**Table 2:** Spearman's Rho correlation between each pair of variables (histological grade vs. RNAScope score; histological grade vs. KIT protein pattern; RNAScope score vs. KIT protein pattern).

	**Histological grade vs. KIT expression pattern**	**Histological grade vs. RNAScope score**	**RNAScope score vs. KIT expression pattern**
**Spearman r**			
r	0.1747	0.7418	0.1545
95% confidence interval	−0.09047 to 0.4168	0.5962 to 0.8402	−0.1111 to 0.3994
***p*** **value**			
p (two-tailed)	0.1818	<0.0001	0.2387
Significant (alpha = 0.05)	No	Yes	No

## Discussion

In the past few years, histopathological techniques for *in situ* analysis of biomarkers such as DNA, RNA, and proteins have been extensively implemented, eventually becoming a routine tool in diagnostic research laboratories for human diseases and translational medicine. In particular, the recent development and availability of RNAScope represented a major improvement over traditional RNA ISH methods that are largely affected by low stability of RNA in the sample (43, 44), often resulting in a suboptimal detection of RNA and also in poor reproducibility (43). In veterinary medicine, the use of RNAScope has begun to emerge, but it is still mainly limited to the detection of specific infectious agents (45, 46) or inflammatory cytokines (47). With the present study, we aimed to experiment and validate the use of RNAScope in detecting and measuring the expression of *c-KIT* mRNA in formalin fixed and paraffin-embedded (FFPE) canine MCTs. In our experiments, we observed that c-KIT mRNA was differently expressed in neoplastic cells according to the histological grade of the tumor, but its expression was overall either comparable with or above the positive PPIB control probe as evaluated in testicular parenchyma or normal skin biopsies, respectively. As expected, *c-KIT* mRNA was normally expressed in Leydig cells and spermatogonia in testicular parenchyma and undetectable in normal skin. As for the normal control probe PPIB, we found a very high expression in testicular parenchyma with a little to moderate expression in normal skin biopsies and no expression in neoplastic cells. These results confirm that TMAs constructed from a retrospective FFPE tissue archive series are fit-for-purpose for the evaluation of *c-KIT* mRNA by RNAScope in canine MCTs; for several reasons, this finding represent a different-making option in biomarker development and discovery. Notably, TMAs give the chance to choose highly representative areas of their donor tissues (36), to test a great number of cases at once, leading to a manifest sparing of reagents and time for the analysis. Furthermore, preserved tissues selected for TMAs construction are often related to long-term clinical follow-up data, hence their fundamental value for the assessment and the evaluation of new prognostic biomarkers. However, we also need to address an important critical issue encountered during our experiments in order to offer useful suggestion to optimize the reproducibility of this assay on FFPE canine MCTs. We had to consider beforehand that canine MCTs are normally accompanied by edema and collagen bundle fragmentation and degeneration; therefore, a prolonged and aggressive treatment with protease could lead to severe artifactual changes. Thus, we decided to slightly modify the standard protocol by reducing protease treatment to 15 min instead of 30 min; this slight modification provided a very good balance between the expression of the RNA probe and the preservation of tissue morphology. The second aim of our work was to apply, for the first time, the RNAScope method to evaluate the association between *c-KIT* mRNA expression, histological grade, and immunohistochemical KIT protein pattern distribution. Several authors have made attempts to validate *c-KIT* mRNA levels as a new biological and prognostic marker. For instance, Turin et al. (22) measured *c-KIT* mRNA in the blood of MCT-affected dogs by quantitative PCR (qPCR) and described lower levels of *c-KIT* mRNA in blood specimens compared to tumor biopsies and a progressive reduction of *c-KIT* mRNA levels between 1 and 3 months after surgery. However, the authors did not observe any correlations between *c-KIT* mRNA in blood and tumor grading, degree of neoplasm differentiation, or clinical prognosis. More recently, Giantin et al. (21) measured *c-KIT* mRNA expression in canine MCTs by quantitative real-time PCR searching for possible correlations with tumor grade, immunohistochemical staining pattern, postsurgical prognosis, and mutations. The results obtained from their work indicated that *c-KIT* mRNA is overexpressed in canine MCT, although the fold variations were not associated with the protein localization or complementary DNA mutations (21). In line with Giantin et al. findings, we also did not observe a significant correlation between *c-KIT* mRNA and KIT immunohistochemical staining pattern, confirming that the level of *c-KIT* mRNA expression is probably independent of protein localization (15, 21), suggesting that *c-KIT* gene regulation may affect both transcriptional and posttranscriptional mechanisms. However, we observed a strong and significant correlation between histological grade and *c-KIT* mRNA expression. In our opinion, this observation supports the value of measuring *c-KIT* mRNA in canine MCT samples, but it should be considered as exploratory and hypothesis generating for the lack of correlation with clinical follow-up data. Further studies are necessary to overcome this limitation, but we believe that the translation of this result in a wider clinical setting would conclusively address the potential value of *c-KIT* mRNA alone, in addition to immunohistochemistry or in combination with other markers, to define a prognosis or to predict response to therapies. The immunohistochemical staining of KIT receptor protein is still considered one of the most useful prognostic parameters in canine MCTs, and numerous studies recently reviewed by Welle et al. (16) and Gil da Costa (17) have shown a strong correlation between altered (cytoplasmic focal or cytoplasmic diffuse) KIT expression and higher histological grade. According to other authors, our results suggest that a non-specific staining and/or discrepancies based on a subjective and semiquantitative interpretation of immunohistochemistry for KIT protein may negatively affect the interpretation of the results, leading to an improper association between KIT protein staining pattern and histological grade (48). Conversely, chromogenic RNA hybridization is confirmed to be a robust, sensitive, and specific technology showing an almost total absence of background and non-specific staining and thus a minimal interobserver variation. “Our findings also showed that *c-KIT* mRNA may be expressed both in cytoplasm and nuclei of neoplastic cells. The presence of nuclear mRNA fraction may possibly be consistent with nuclear retention of c-KIT transcripts as the result of an inefficient regulation of gene expression. Several authors have suggested that nuclear mRNA retention and compartmentalization may be considered as an important mechanism regulating the activity of transcription-related proteins and modulating cell growth and death (49). According to other authors (50–52), our results allow us to speculate that mRNA transcription is less efficient in neoplastic cells for the presence of specific genetic or epigenetic alteration. The disturbance of the delicate equilibrium between nuclear mRNA retention and its decay could potentially lead to the persistence of RNA transcript that are non-functional or potentially deleterious for the cells (49–52). Although scientifically tempting, the investigation of concurrent and specific *c-KIT* mutations wasn't considered for this study. It has been described that overexpression of *c-KIT* mRNA, increased KIT expression or aberrant KIT protein localization may be independent from mutations of *c-KIT* (21). However, we believe that further dedicated researches are necessary to elucidate *c-KIT* mRNA transcription dynamics. The unveiling of the genetic and the epigenetic mechanisms underlying canine MCTs will hopefully help the development of specific targeting therapies.” In summary, we have developed a manual version of the RNAScope technology for the assessment of *c-KIT* mRNA in canine MCTs. This technology may be tested and validated both for research and for the clinical practice. Our results also demonstrated a correlation between *c-KIT* mRNA expression and histological grade, but further investigations are needed to confirm these findings that may potentially have an important significance for prognosis and treatment of canine mast cell tumor.

## Data Availability Statement

The raw data supporting the conclusions of this article will be made available by the authors, without undue reservation.

## Author Contributions

DDB drafted the manuscript and contributed to the study concept, study design, and analysis and interpretation of data. DDB, FP, GP, Id'A, AI, and FZ conducted the histopathological and immunohistochemical analyses and RNAScope. FZ and VB provided technical and scientific support. OP, RF, DDB, GP, and FP revised the manuscript for content and contributed to the interpretation of data. OP also contributed to the study concept and design. All authors contributed to the article and approved the submitted version.

## Conflict of Interest

The authors declare that the research was conducted in the absence of any commercial or financial relationships that could be construed as a potential conflict of interest.

## Supplementary Material

Supplementary Figure 1 Morphologic features of Canine Mast Cell Tumors. According to the Kiupel two-tier grading system, low grade canine cutaneous MCTs were composed of relatively monomorphic populations of neoplastic mast cells filled with basophilic granules and that have a low nuclear to cytoplasmic ratio, minimal anisokaryosis, single nuclei, often with only one to two nucleoli, and a low mitotic index. Collagen oedema, flame figures and eosinophils infiltration were also seen **(A–C)**. **(D)** High grade canine MCTs showed several atypical mitosis (arrowhead) and at least three cells with three or more nuclei (arrows) in 10 HPF. Variation of nuclear diameters (karyomegaly and anisokaryosis) was also observed at least two times in at least 10% of neoplastic cells. Haematoxylin and Eosin stain, original magnification 10x for **(A)** and 40x for **(B–D)**.
