# Supplementary figures and images for: RNAScope in situ Hybridization as a Novel Technique for the Assessment of c-KIT mRNA Expression in Canine Mast Cell Tumor
Source: Front Vet Sci. 2021 Feb 16;8:591961. doi: 10.3389/fvets.2021.591961 (PMC7921150; doi:10.3389/fvets.2021.591961)

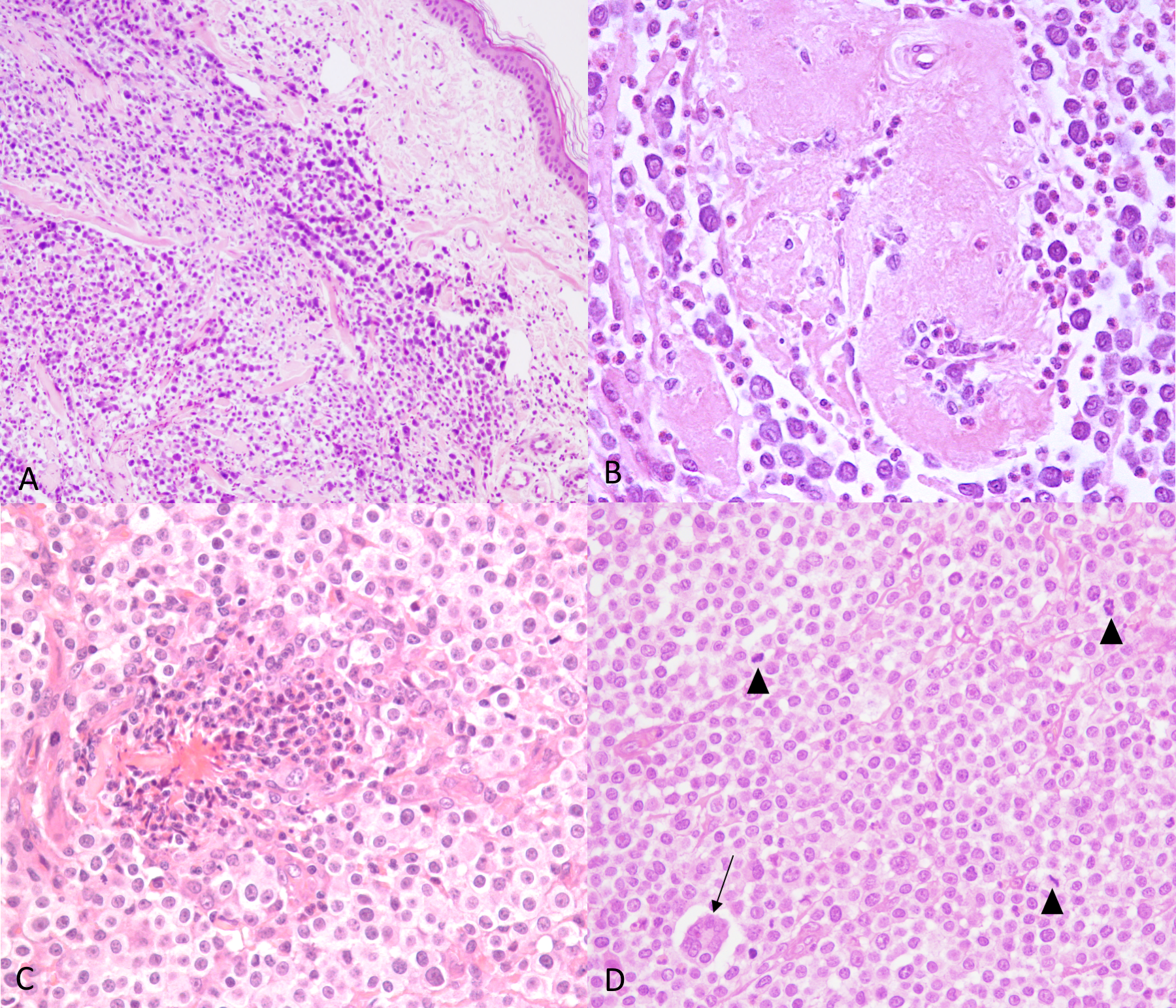

Supplement: Supplementary Figure 1 — Morphologic features of Canine Mast Cell Tumors. According to the Kiupel two-tier grading system, low grade canine cutaneous MCTs were composed of relatively monomorphic populations of neoplastic mast cells filled with basophilic granules and that have a low nuclear to cytoplasmic ratio, minimal anisokaryosis, single nuclei, often with only one to two nucleoli, and a low mitotic index. Collagen oedema, flame figures and eosinophils infiltration were also seen (A–C). (D) High grade canine MCTs showed several atypical mitosis (arrowhead) and at least three cells with three or more nuclei (arrows) in 10 HPF. Variation of nuclear diameters (karyomegaly and anisokaryosis) was also observed at least two times in at least 10% of neoplastic cells. Haematoxylin and Eosin stain, original magnification 10x for (A) and 40x for (B–D). [file Image_1.TIFF]
